# Nugget Formation and Mechanical Behaviour of Friction Stir Welds of Three Dissimilar Aluminum Alloys

**DOI:** 10.3390/ma13112664

**Published:** 2020-06-11

**Authors:** Neves Manuel, Ivan Galvão, Rui M. Leal, José D. Costa, Altino Loureiro

**Affiliations:** 1 CEMMPRE, Departamento de Engenharia Mecânica, Universidade de Coimbra, Rua Luís Reis Santos, 3030-788 Coimbra, Portugal; ivan.galvao@dem.uc.pt (I.G.); rui.leal@dem.uc.pt (R.M.L.); jose.domingos@dem.uc.pt (J.D.C.); altino.loureiro@dem.uc.pt (A.L.); 2 Escola Superior Politécnica do Namibe, Rua Amílcar Cabral, Moçâmedes 201, Angola; 3 ISEL, Departamento de Engenharia Mecânica, Instituto Politécnico de Lisboa, Rua Conselheiro Emídio Navarro 1, 1959-007 Lisboa, Portugal; 4 LIDA-ESAD.CR, Instituto Politécnico de Leiria, Rua Isidoro Inácio Alves de Carvalho, 2500-321 Caldas da Rainha, Portugal

**Keywords:** friction stir welding, three dissimilar aluminum alloys, welding speed, T-joints, microstructure, mechanical properties

## Abstract

The aim of this research was to investigate the influence of the properties of the base materials and welding speed on the morphology and mechanical behavior of the friction stir welds of three dissimilar aluminum alloys in a T-joint configuration. The base materials were the AA2017-T4, AA5083-H111, and AA6082-T6 alloys in 3 mm-thick sheets. The AA6082-T6 alloy was the stringer, and the other alloys were located either on the advancing or retreating sides of the skin. All the T-joint welds were produced with a constant tool rotation speed but with different welding speeds. The microstructures of the welds were analyzed using optical microscopy, scanning electron microscopy with energy dispersive spectroscopy, and the electron backscatter diffraction technique. The mechanical properties were assessed according to micro-hardness, tensile, and fatigue testing. Good quality welds of the three dissimilar aluminum alloys could be achieved with friction stir welding, but a high ratio between the tool’s rotational and traverse speeds was required. The welding speed influenced the weld morphology and fatigue strength. The positioning of the skin materials influenced the nugget morphology and the mechanical behavior of the joints. The joints in which the AA2017 alloy was positioned on the advancing side presented the best tensile properties and fatigue strength.

## 1. Introduction

Aluminum alloys of the 5xxx, 6xxx, and 2xxx series are widely used in various industrial sectors, such as shipbuilding, aerospace, building structures, bridge structures, and even military vehicles, due to their lightness, mechanical strength, and resistance to corrosion [1,2]. T-joints are currently used in these industries to increase the stiffness of thin plates.

Friction stir welding (FSW) is a solid-state welding process that prevents porosity and cracking, but the formation of defects is influenced by the flow of materials around a tool. The flow of material in this zone depends on the tool’s geometry, the tool’s rotation speed and welding speed, the tool’s axial force or displacement, and even the tool’s tilt angle, whether in similar or dissimilar material welds [3,4]. Fratini et al. [5], when comparing similar welds for the AA6082-T6 and AA2024-T4 alloys in a T-joint configuration, concluded that the temperature fields and strain rate influence the flow of a material in the stir zone and, hence, the integrity of welds.

In welds between dissimilar materials of different families, the properties of the base materials have to be considered because of the formation of intermetallic compounds that have very different physical and mechanical properties from the base materials and influence the flow of materials in the stir zone [6,7]. However, even for dissimilar welds of materials within the same family, the influence of the properties of the materials on the formation of the weld can be significant. Silva et al. [8] stated that in the dissimilar butt welds of AA7075-T6 to AA2024-T3, the mixing of the two materials in the stir zone was greatly influenced by the geometry and rotational speed of the tool, only obtaining satisfactory mixing at high rotational speeds.

Dinaharan et al. [9] stated that the material that is on the advancing side occupies most of the stir zone and influences the strength of butt weld in aluminum 6061 in rolled and cast plates. Barbini et al. [10] showed that, for butt welds between AA2024-T3 and AA7050-T7651, a better material flow in the stir zone is obtained when the AA2024-T3 is located on the advancing side. Better material flow in the stir zone was also observed for butt welds between AA2024-T6 and AA6061-T6, when the latter material was placed on the advancing side [11]. The results mentioned above suggest that the flow of materials in the stir zone depends on the mechanical properties of the materials on the advancing and retreating sides. Manuel et al. [12] also stated that for T-joints between AA6082 and AA5083, material flow and defect formation are greatly influenced by the joint type, tool geometry, and process parameters, as well as the materials’ properties. The investigations found in the literature about three dissimilar aluminum alloys only concern lap joints [13,14].

The FSW of three dissimilar aluminum alloys has scientific and industrial interest, since there is a need for new combinations of materials and there is no clear understanding of how material properties influence weld formation. The aim of this study was to analyze the influence of the properties of the base materials and the variation of the welding speed on the morphology and mechanical properties of welds of three dissimilar aluminum alloys arranged in a T-joint configuration.

## 2. Materials and Methods

The experimental tests were performed on 3 mm-thick sheets of AA2017-T4, AA5083-H111, and AA6082-T6 aluminum alloys. Alloys that require plastic deformation during construction are used without much hardening, as in the case the alloys 5083-H111 and 2017-T4, while reinforcements are made of hardened alloys, as is the case for 6082-T6. The chemical composition and mechanical properties of these alloys are listed in Table 1 and Table 2, respectively.

**Figure 1:**
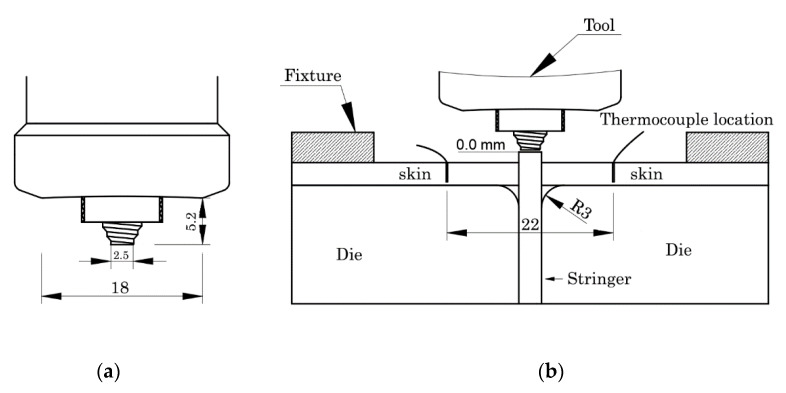
(**a**) Geometry of the tool; (**b**) welding setup and starting point to measure tool penetration.

A tool of H13 quenched and tempered steel, composed of a progressive (cylindrical and conical) threaded pin of 5.2 mm in length and a shoulder of 18 mm in diameter with a concavity of 5° (see Figure 1a) was used. Previous tests have shown that this tool geometry has obtained defect-free welds in dissimilar aluminum alloys [12].

The setup used to perform the welds is shown in Figure 1b. The stringer protruded 1.4 mm in order to provide enough material to fill the empty volumes between plates and dies in the fillets. The AA6082 alloy was the stringer for all the weld series. The AA5083 and AA2017 alloys were positioned on the advancing and retreating sides of the skin, respectively, in some series—they were designated as 562 and the opposite in another series, designated as 265. The dimensions of the stringer and skin plates were 330 × 37.4 × 3 and 330 × 80 × 3 mm, respectively. The oxides were removed from the interfaces by sanding, and the plates were cleaned with alcohol just before the welding.

The welds were performed in position control using a Cincinnati Milacron 207 Mk milling machine, and the tool’s rotational speed (w-500 rpm), plunge depth (7.1 mm), and tilt angle (3°) were maintained constant for all series. The tool’s welding speed (v) was changed according to Table 3. The weld series designation consisted of the designation of the sequence of the materials, followed by the welding speed. The parameters were chosen based on previous experience. The (*w/v*) ratio is also indicated in Table 3 due to its relationship with the heat input, which increases with the ratio [15].

During welding, the thermal cycles were measured by k-type thermocouples embedded in small holes close to the shoulder’s trajectory, on the advancing and retreating sides; see Figure 1b. A data translation device with an acquisition rate of 75 Hz and cold junction compensation was used to record the thermal cycles.

After welding, 63 × 25 × 25 mm specimens were transversely removed to the welding direction of all the series, ground down with sandpaper P2500, and then polished using 3 and 1 µm diamond suspensions. Modified Keller’s reagent was effective in revealing the grain boundary of AA2017 and Weck’s reagent was effective for such for AA6082, but no effective reagent was found for AA5083. The grain size was determined by the Heyn intercept method. A Leica DM4000M LED optical microscope was used to analyze the samples. In order to better identify which of the alloys was present in each zone of the nugget, semi-quantitative chemical analyses were performed by scanning electron microscopy/energy dispersive spectroscopy (Zeiss, MERLIN, Field Emission Scanning Electron Microscope-Gemini II/Oxford Instruments, X-MAXN) at 10 kV, bearing in mind that the AA2017 alloy was Cu-rich, the AA5083 alloy was Mg-rich and the AA6082 alloy had intermediate contents of Mg and Si. The samples for electron backscatter diffraction (EBSD) analysis were grinded and polished with 3 and 1 μm diamond suspensions and finished with colloidal silica. Finally, an electrolytic polishing was performed by using a solution of nitric acid and methanol at −10 °C and 15–20 V for 30 s. EBSD was performed with an FEI QUANTA 400F scanning electron microscope provided with an EBSD detector system using a scan step size of 0.65 μm.

The Vickers microhardness profiles of the welds were determined using an HMV-G SHIMADZU tester on the weld’s cross-section, using 200 g for 15 s. The distance between the test points was 0.5 mm in the nugget zone and 1 mm in the heat-affected zone (HAZ) and base materials.

The base materials were tensile tested at room temperature and at high temperatures (320 and 450 °C) using loading speeds of 2 and 72 mm/min, respectively on an Instron 4206 machine provided with a three-stage oven. Three specimens were tested for each base material.

**Figure 2:**
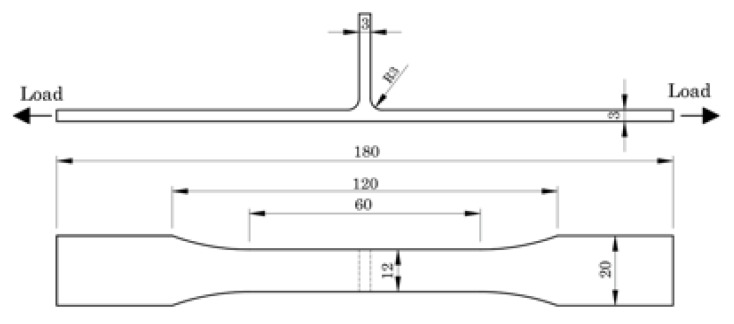
Fatigue specimen—shape and size.

The welded specimens for the tensile and fatigue tests were cut transversely to the direction of the weld with the dimensions of 180 × 20 mm (length × width). The shape and size of the fatigue specimens are shown in Figure 2. The edges of the specimens were rounded and polished to avoid a concentration of surface stresses and the initiation of cracks. The tensile tests were performed in accordance with the ASTM E8 standard for testing metallic materials [16], and the loads were applied in the direction of the skin. Three tensile specimens were tested for each weld series. The local strain fields were recorded with an ARAMIS 3D 5 M optical extensometer from GOM GmbH with digital image correlation (DIC). This is a real time system of measurement of 3D surface strains, based on triangulation, that employs image registration and the tracking of changes in images over time. The specimens were prepared by applying a random black speckle pattern over the previously mat white-painted side surface.

The fatigue tests were carried out using an Instron servo-hydraulic machine coupled to an Instron Fast Track 8800 acquisition and control system. The range of stresses varied between 150 and 200 MPa with a frequency of 15–25 Hz; the frequency decreased as the maximum load applied increased, and the stress ratio was set to 0.02. Two test pieces were used for each test condition. In cases where there was greater dispersion, a third trial was carried out. The fracture surface of the fatigue specimens was studied using a Zeiss, MERLIN, field emission scanning electron microscope.

## 3. Results and Discussion

### 3.1. Thermal Cycles in the Welds

The formation of defects is currently attributed to insufficient heat generation in the weld, inadequate material flow around the pin, and the insufficient consolidation of the deformed material at the back of the pin, all of which are factors controlled by welding parameters [17]. For this reason, the influence of some process parameters on the thermal cycles induced in the welds was analyzed.

**Figure 3:**
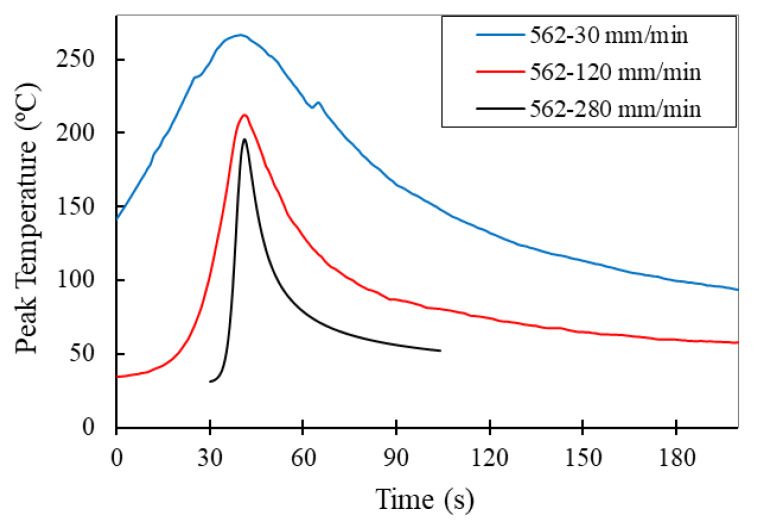
The thermal cycles measured in the 562 series, as performed using different welding speeds.

Figure 3 shows the thermal cycles measured on the advancing side for the 562 series. As the welding speed of the series increased, the peak temperature and the cooling cycle time decreased due to the lower ratios (*w/v*); see Table 3.

**Figure 4:**
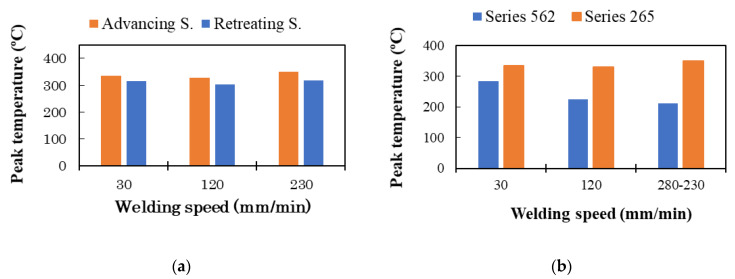
Peak temperature measured (**a**) on the advancing and retreating sides of the weld series 265 and (**b**) on the advancing side of the 562 and 265 weld series.

Though these thermal cycles were recorded far from the center of the nugget (11 mm), the curves illustrate the influence of the welding speed on the heat input during the process. On the other hand, the maximum temperature reached in the welds was higher on the advancing side than on the retreating side by about 27 °C, as illustrated in Figure 4a. This was due to the asymmetry of heat generation around the tool, as has been shown by other researchers [18,19]. The induced thermal cycle was also greatly influenced by the materials that were located on the advancing and retreating sides. Figure 4b shows that the measured peak temperature was higher when AA2017 was on the advancing side than when it was on the retreating side. This means that there was a larger energy consumption to plastically deform this material than AA5083, so the material position had a more significant effect on the heat generated than the asymmetry of the process.

Figure 4b also shows that the peak temperature observed for the 265-230 series was higher than that obtained in the 265-120 series; this was due to a slight offset of the tool closer to the thermocouple on the advancing side in the 265-230 series.

### 3.2. Morphology of the Welds

**Figure 5:**
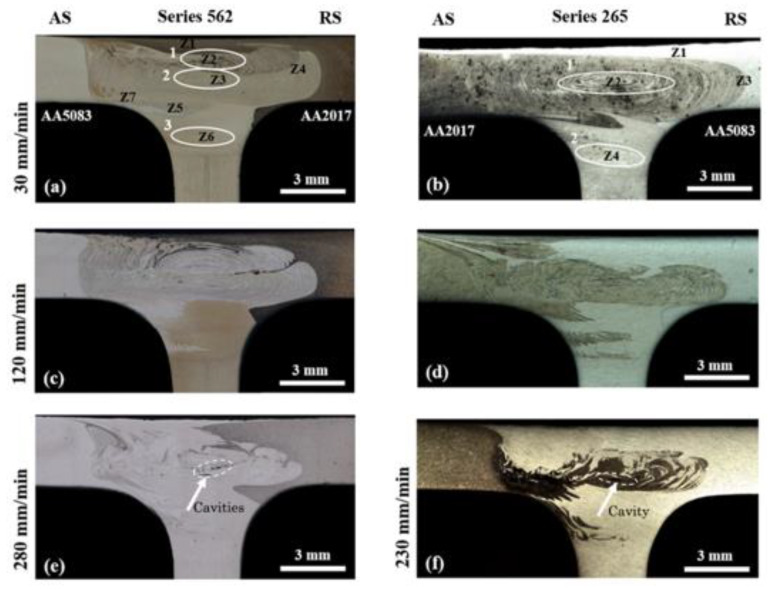
Cross-section macrographs of the three dissimilar materials weld series: (**a**) 562-30, (**b**) 265-30, (**c**) 562-120, (**d**) 265-120, (**e**) 562-280, and (**f**) 265-230.

All welds showed good surface appearance; however, the differences mentioned in the thermal cycles should have influenced the formation of the weld nugget. Figure 5 illustrates the cross-sectional macrographs of the 562 and 265 weld series. This image shows that all the welds had well-defined radii of the fillets, which indicates the effectiveness of the welding parameters and the adopted T-joint configuration. The points where chemical analysis (EDS) was performed are marked with numbers in these macrographs, e.g., Z1, Z2, and Z3, to better aid the interpretation of the flow of the three materials in the nugget. The chemical composition of the different zones is indicated in Table 4.

An analysis of the macrographs revealed differences in nugget morphologies that varied with increasing welding speeds, as well as with the position of the base materials on the advancing or retreating side. It was possible to observe a great asymmetry in the flow of materials in all the macrographs in relation to the welding center line in both the 562 and 265 series.

The 562-30 series featured three onion ring structures—two located in the skin and one in the stringer fillet zone (marked with ellipses 1, 2, and 3, respectively)—and other zones with different colors; see Figure 5a. Zone 1 essentially consisted of AA2017 positioned on the rear side, which was dragged by the shoulder and remained at the top of the nugget, as seen in Table 4. This table shows only some of the measurements in the 562-30 and 265-30 series in order to illustrate the main differences and reduce the size of the article. The onion ring structure just below ellipse 1—zone 2—was essentially composed of interspersed layers of the three alloys; the dark layers consisted of AA2017. The second onion ring structure indicated by ellipse 2—zone 3—had interleaved layers composed of the three alloys, varying in composition with the location, but with less contribution from AA2017; see Table 4. In zone 4, there was a really good contribution from AA5083. Zone 5 consisted of AA5083 coming from the advancing side. While the material flow on top was a shoulder-driven flow, the last flows were, in fact, pin-driven flows. The onion ring structure was composed of the three materials on the advancing side, as illustrated by zone 7. This showed a good mix of the three materials in the stir zone, contrary to what has been suggested for dissimilar welds [20].

Zone 6 corresponded to the stringer region next to the advancing side fillet, influenced by the action of the pin tip. There was the formation of an onion ring structure, resulting from the contribution of only AA6082 and AA2017. This showed that only one material of the skin, the AA2017, flowed downward into the fillet zone between the tool and dies, which is contrary to what was suggested by Manuel et al. [12] for dissimilar welds.

For the 265-30 series, only one onion ring was formed on the skin and another was formed on the stringer fillet zone, as indicated by ellipses 1 and 2 in Figure 5b. The top layer in this weld consisted of AA6082 (see zone 1), as opposed to the 562-30 series, which had AA2017 on the top; see Table 4. Zone 2 consisted of interspersed layers of the skin materials AA2017 and AA5083. The AA6082 alloy also probably coexisted in this zone, but it was difficult to distinguish via the chemical composition analysis. The peripheral zone of the nugget on the retreating side consisted of layers of only AA5083 and AA2017, according to zone 3. The onion ring structure in the fillet (zone 4) consisted of interleaved layers composed of AA6082 and AA2017, with no AA5083.

**Figure 6:**
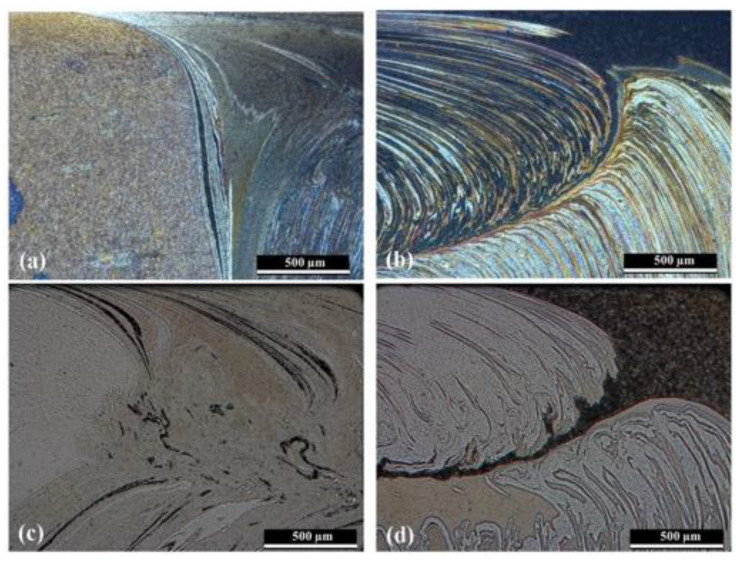
Comparison of material flow between the advancing and retreating sides of 562 welds series: (**a**) Advancing Side-562-30, (**b**) Retreating Side-562-30, (**c**) Advancing Side-562-120, and (**d**) Retreating Side-562-120.

As the welding speed increased, in the 562-120 series, the onion ring structure on the fillet tended to disappear, although the material flow remained in this zone; see Figure 5c. In addition, the two onion ring structures in the skin tended to separate, with material from the retreating side entering between the onion rings. This is more visible in Figure 6, where the morphology of the material flow from the 562-30 and 562-120 weld series is compared at a higher magnification. Figure 6a,c compares the advancing sides and the retreating sides (Figure 6b,d).

For the 265-120 series (Figure 5d), the onion ring structure tended to fade, revealing a lack of time or an inability for an orderly flow of layered materials but with increased participation of the AA2017 alloy. This suggested that this alloy had a greater capacity for hot plastic flow and a greater need for time and/or temperature for the AA5083 alloy to flow in layers when it was located on the retreating side.

The onion ring structures disappeared in the 562-280 and 265-230 series, and the stir zone presented a more chaotic appearance with the formation of small internal cavities, either in the center or in the advancing side, as marked with arrows in Figure 5e,f. This was because the heat input decreased as the *w/v* ratio decreased, so less material was dragged by the tool and there was less time and temperature for the stable and periodic flow of materials (as suggested by Yoon [21]), thus causing the formation of defects.

Furthermore, it was found that the AA5083 alloy never went downwards to the stringer, regardless of its advancing or retreating side position. This showed that the formation of the nugget depended not only on the position of the materials and process parameters but also on their intrinsic ability to deform at a high temperature.

**Figure 7:**
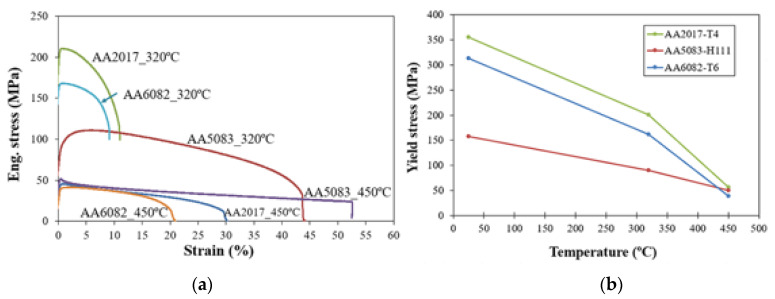
Variation of the mechanical properties of base materials with temperature: (**a**) Engineering tensile stress–strain curves and (**b**) yield stress curves.

Figure 7a shows the engineering tensile stress–strain curves of the three base materials obtained at temperatures of 320 and 450 °C, as well as the variation of the yield stress with temperature; see Figure 7b. The alloys still had very different yield stresses and tensile strengths at 320 °C, and AA2017 had the highest values. The AA2017 and AA6082 alloys showed significant work softening soon after reaching yield stress, unlike the AA5083 alloy that hardened until a strain of 5% before starting to soften. In turn, the AA5083 alloy exhibited much greater plastic strain at fracture than the AA2017 and AA6082 alloys, probably due to dynamic recovery, as suggested by Shi et al. [22]. At 450 °C, a noticeable loss of mechanical strength was observed for all the alloys, as was an obvious increase in strain at fracture. Some dissolution and coarsening of strengthening precipitates and partial recrystallization may explain the loss of strength and the increased ductility of the heat-treatable alloys (AA6082 and AA2017) [22,23]. In addition to the loss of strength, the AA5083 alloy had a large steady state flow due to dynamic recrystallization [24]. For this temperature, the yield stress of this alloy was higher than that of AA6082; see Figure 7b.

The heated material was mainly exposed to compression and shear stresses in the stir zone due to its complex interaction with the tool and the colder surrounding material [25]. Though the tensile behavior of the materials at high temperatures did not match what happens in welding, it helped to understand the material flow and was easier to obtain. In welding, temperature decreases rapidly as the distance to the tool increases, so, for the AA5083 alloy, it should be difficult for the tool to drag the material in this zone because some work hardening occurs at a low temperature. Therefore, a little volume of material is dragged around the pin, which results in the poor weldability of the alloy. This is compatible with the difficulty for the AA5083 alloy to flow downwards from the skin to the stringer, which was observed in the welds presented above. The AA6082 and AA2017 alloys experienced significant temperature softening, which allowed the AA2017 to flow downwards from the skin to the stringer, as illustrated above. Similar behavior was observed by Leitão et al. [26] for the AA6082 and AA5083 alloys used in butt welds. The results presented above suggest that, for this welding process, the AA2017 and AA6082 alloys show better weldability than the AA5083.

### 3.3. Microstructure

**Figure 8:**
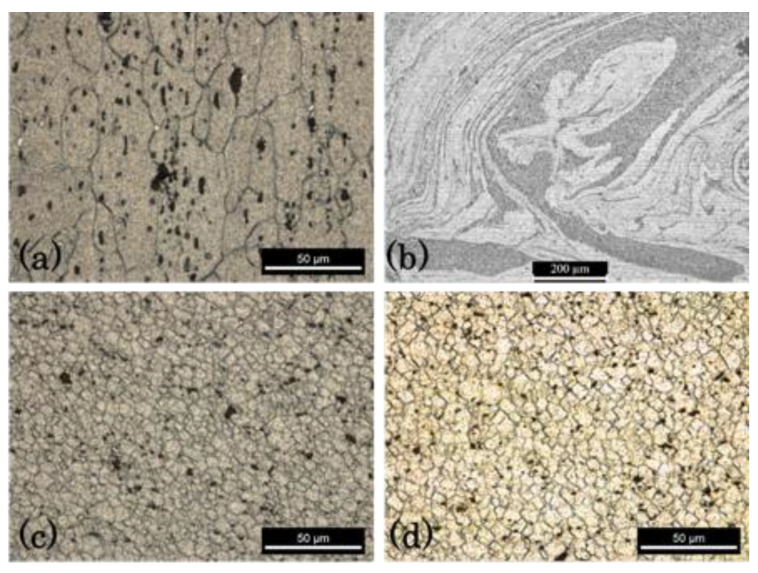
Microstructure of the: (**a**) base material AA 6082, (**b**) nugget of the 562-120 series, (**c**) nugget of the 562-30 series, and (**d**) nugget of the 265-30 series.

The microstructures in the nugget of the welds between the three dissimilar Al alloys were very complex and difficult to uncover, especially when taking the definition of the grain boundary into consideration. The etchants for each material were different and did not always work when the materials were together. Figure 8a shows a micrograph of the AA6082 base material etched with Weck’s reagent. The material was composed of grains elongated in a rolling direction with an average grain size of 59 × 26.3 µm. The distribution of each material in the nugget was very complex, as is illustrated in Figure 8b, for a 562-120 weld. This image shows, at a smaller magnification, a refined grain structure in the areas where AA6082 was present. In the surrounding areas, which were made up of the other materials, the grain boundary was not revealed with this etchant. Figure 8c,d illustrates the microstructure of the same alloy in the nuggets of the 562-30 and 265-30 weld series, respectively. When comparing these last two figures with Figure 8a, a marked refinement of the grain in the nugget is visible. The difference in the average grain size in the nugget between the 562-30 and 265-30 series was marginal, as the grain sizes were about 5.1 ± 1.5 and 5.2 ± 1.6 µm, respectively. A large standard deviation was observed, because the grain size varied with the location. According to the thermal cycles illustrated in Section 3.1, a larger difference in grain size from both weld series would be expected. A possible cause of this similarity was that most of the grain sizes were measured in the central nugget or even on the retreating side, where the grains were most visible.

Increasing the welding speed—from 30 to 280 mm/min, for instance—had a very small effect on the nugget grain size, with it remaining within the range of 4–5 µm. For AA2017, although its grain size (20.2 × 9.5 µm) was smaller than that of AA6082, the nugget grain size was also in the 4–5 µm range for all the welding series. The significant plastic deformation suffered by the materials in the stir zone and the recrystallization occurring in small and very confined areas, as seen in Figure 8b, could penalize grain growth with the ephemeral increase in heat input. The authors believe that the coexistence of the three alloys in the nugget cancelled the effect that the increase in welding speed had on the decrease in grain size, contrary to that suggested by Kalemba-Rec et al. [27]. A study by Ahmed et al. [28] found that grain size decreases with the increase in welding speed for similar welds, but the same effect does not occur for dissimilar welds.

**Figure 9:**
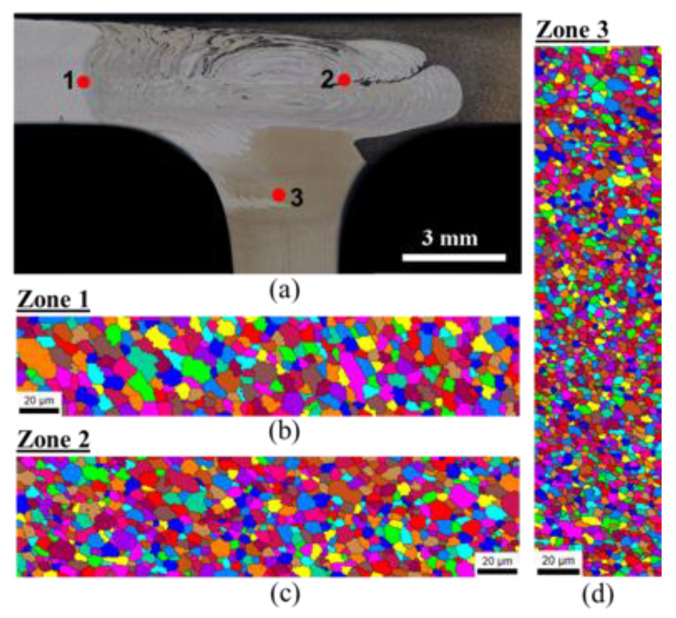
Electron backscatter electron backscatter diffraction (EBSD) analysis conducted for the 562-120 weld: (**a**) zones analyzed and EBSD maps registered in zones 1 (**b**), 2 (**c**), and 3 (**d**).

**Figure 10:**
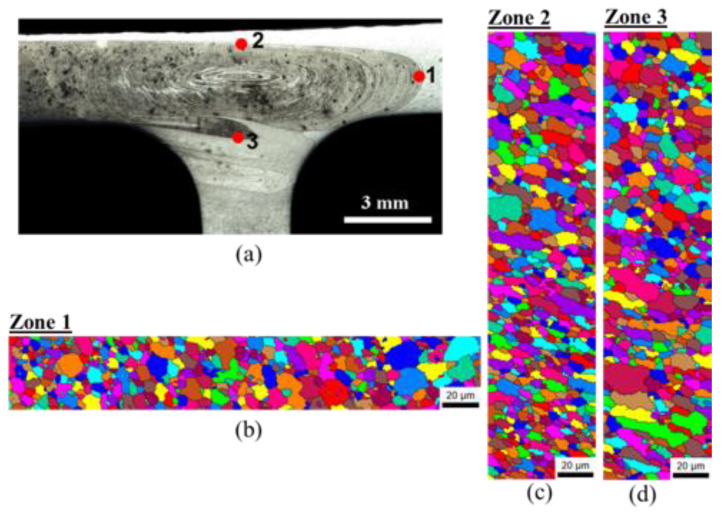
EBSD analysis conducted for the 265-30 weld: (**a**) zones analyzed and EBSD maps registered in zones 1 (**b**), 2 (**c**), and 3 (**d**).

The nuggets of both weld series were analyzed by EBSD to study the weld microstructure further, as shown in Figure 9 and Figure 10 for the 562 and 265 welds, respectively. The regions under analysis are indicated by dots in Figure 9a and Figure 10a to make them more discernible in the weld macrographs, although they are small vertical or horizontal scanning lines. It can be observed from the figures that the onion-ring regions of both welds tended to present a refined microstructure, which agreed well with the metallographic study, but the grain size was not uniform in the nugget. A gradient in grain structure is visible in these maps, with the grains composing the onion-ring regions presenting a smaller grain size and being more equiaxed than the grains in neighboring areas; see Figure 9b,c and Figure 10b–d. Plastically deformed grains are visible both in the onion-ring regions and the neighboring areas (see Figure 9b and Figure 10c), which agrees well with the flow features characterized above. A further refined microstructure (2 µm) is also observed in Figure 9d, and this corresponds to a region in the stringer that was under the action of the pin tip where little heat was generated. The great microstructural heterogeneity of the weld nugget was due not only to the differences in local heat generated but also to the very complex flow features of the different alloys that made it up.

### 3.4. Hardness and Tensile Behavior

**Figure 11:**
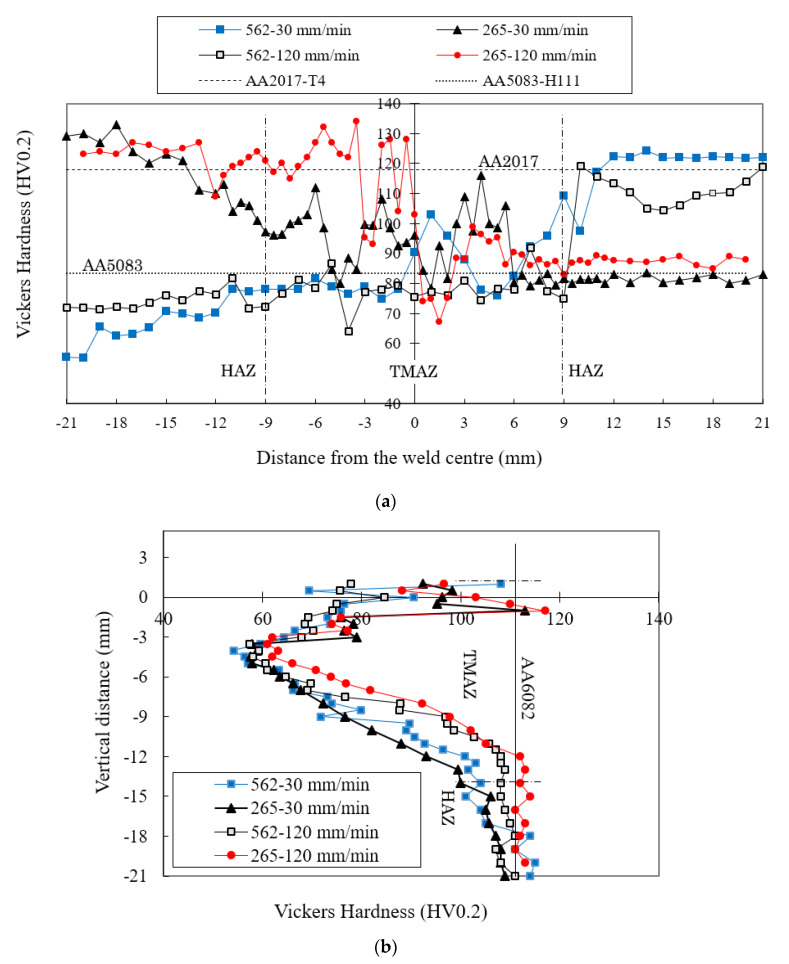
Effect of variation of the welding speed on the hardness profile in the (**a**) skin and (**b**) stringer.

The skin hardness profiles of the 562 and 265 weld series are shown in Figure 11a for defect-free welds. The hardness of the base materials is also shown in the same figure using dashed (AA2017) and dotted (AA5083) lines. It appears that the increase in welding speed did not significantly change the hardness in the HAZ, either on the AA5083 or AA2017 sides. However, there was a slight loss in hardness in the HAZ close to the tool path, mainly when AA2017 was located on the advancing side for the lowest welding speed. This could be attributed to the dissolution of hardening precipitates, as stated by Dong et al. [29]. The stir zone had an irregular hardness pattern with some peaks in the current welds as a result of the non-homogeneous mixing of the three different base materials during the process, as illustrated in Section 3.2.

The stringer hardness profiles for the 562 and 265 series are shown in Figure 11b. In all the series, there was a significant reduction in hardness in the thermomechanically-affected zone (TMAZ) and HAZ, which was generally attributed to the dissolution and coarsening, respectively, of the hardening precipitates [29]. This reduction in hardness was higher for the lowest welding speed series due to the higher and longer thermal cycles, as per Section 3.1.

**Figure 12:**
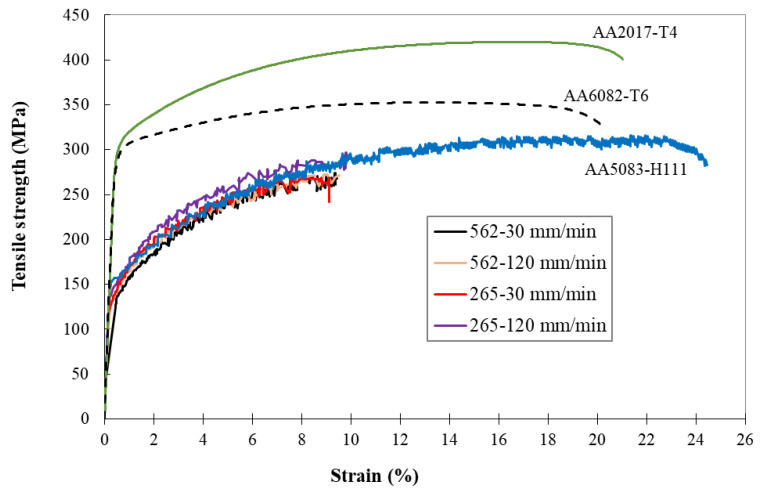
Tensile stress–strain curves of the 562 and 265 weld series and the base materials.

Figure 12 shows the tensile curves of the skin of defect-free specimens of the 562 and 265 series, as well as of the three base materials for comparison. Table 5 summarizes the mean values of tensile strength and strain at failure of each series, as well as the zones where the failure occurred. The effect of welding speed on weld strength is well-illustrated in Figure 12, where the welds performed at the highest speed were slightly above the others but just below the AA5083 alloy.

**Figure 13:**
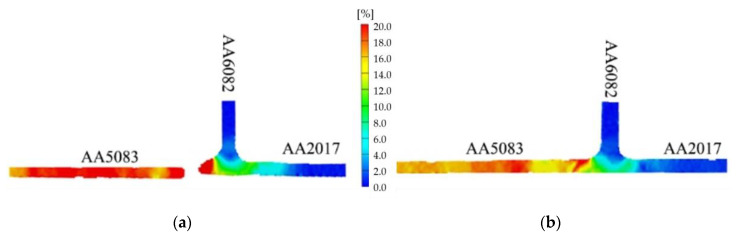
Strain distribution in tensile test specimens close to failure from the (**a**) 562-30 and (**b**) 562-120 series.

Table 5 shows that specimens from both series (562 and 265) made at the lowest welding speed (30 mm/min) had the lowest efficiency values (losses of about 20%) and broke in the HAZ, close to the stir zone, on the AA5083 side. The efficiency is defined as the ratio between the maximum tensile strength of each weld series and the tensile strength of the least resistant base material, in this case being AA5083. Specimens from both series performed at 120 mm/min had the highest efficiency values, as suggested by the hardness results, although the difference was small; see Figure 11a. In addition, these test specimens broke in the HAZ on the AA5083 side but far from the stir zone. This is illustrated in Figure 13, which represents the distribution of the strain fields, obtained by an optical extensometer in two specimens produced with different welding speeds, and at an instant close to failure. Figure 13a illustrates a specimen from the 562-30 series whose highest plastic deformation, the red zone, and failure occurred in the HAZ next to the stir zone, while Figure 13b illustrates a specimen form the 562-120 series that also broke in the HAZ but far from the stir zone.

**Figure 14:**
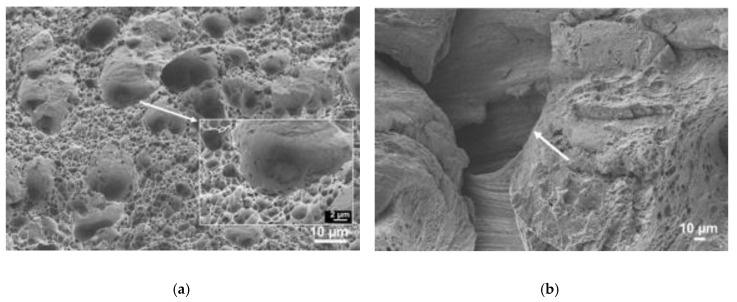
Fracture surface morphology of a specimen in the (**a**) 562-30 and (**b**) 562-280 series.

Figure 14 illustrates the influence of the welding speed on the fracture surface morphology of test pieces from series 562-30 and 562-280 performed at speeds of 30 and 280 mm/min, respectively. Figure 14a shows a ductile fracture surface of a 562-30 specimen with thin dimples and some larger dimples, as shown in the enlargement (5000x) of a zone in the lower right corner of the image. The fractures of the remaining series had similar morphologies because they occurred in the HAZ of the same alloy. Figure 14b shows a ductile fracture surface of a 562-280 series specimen, but the fracture was caused by a defect here, already mentioned in Section 3.2 and indicated by an arrow in the image.

### 3.5. Fatigue Strength

**Figure 15:**
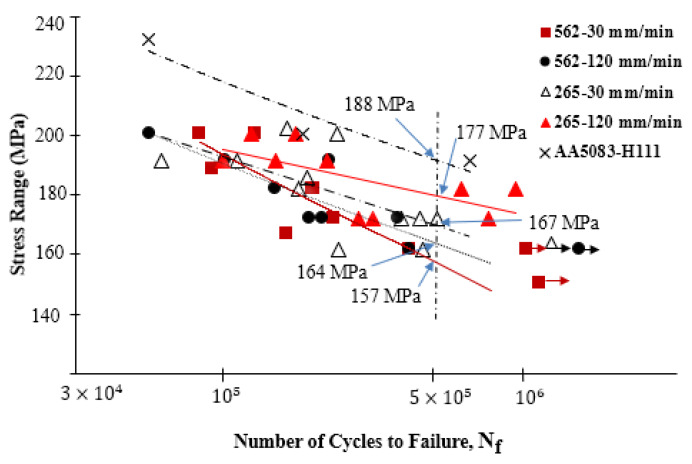
Influence of welding speed on the fatigue strength of the 562 and 265 series.

Figure 15 shows the S–N (stress range/number of cycles to failure) curves for the 562 and 265 series made at 30 and 120 mm/min, as well as the curve for the AA5083 alloy (the least resistant of the base materials). The fatigue test specimens that did not break for more than one and a half million cycles are shown with a horizontal arrow.

This figure shows that all the weld series presented a lower fatigue strength than the base material, which indicates that the welding process reduced fatigue strength regardless of the welding parameters used. However, the 562-120 series, produced at the advancing speed of 120 mm/min, presented better resistance to fatigue compared to the 562-30 series, performed at a lower speed of 30 mm/min but with an increase in the fatigue strength of only 4.5% at 5 × 10^5^ cycles. 

A similar increase in the fatigue strength with the increase in the welding speed could be observed in the 265 series for identical welding speeds of about 5.6%. Ericsson and Sandstrom [30] observed that for low welding speeds, their welds using the AA6082 alloy showed a slight increase in mechanical strength and fatigue compared to the higher welding speed due to the increased amount of heat generated in welding per unit length. In the current study, the opposite behavior was observed, which can be explained by the increase in mechanical strength with the increase in the welding speed, as shown in Table 5.

Figure 15 further shows that the 265 series had a slightly higher fatigue strength than the 562 series, while the 265-120 series had the highest strength. This may also have been related to the tensile properties of the welds, as specified in Table 5. 

One factor that influenced the fatigue strength had to do with the surface finish of the samples tested, as the presence of small surface defects made crack initiation and propagation more probable, thus leading to premature failures [31]. The specimens were polished before testing in the current work; however, the welding crown was more difficult to polish without significantly reducing the thickness of the skin, which introduced some variability in the results. The specimens broke mostly at the TMAZ or close to the HAZ in the AA5083 alloy, either in the higher or lower stress ranges. 

**Figure 16:**
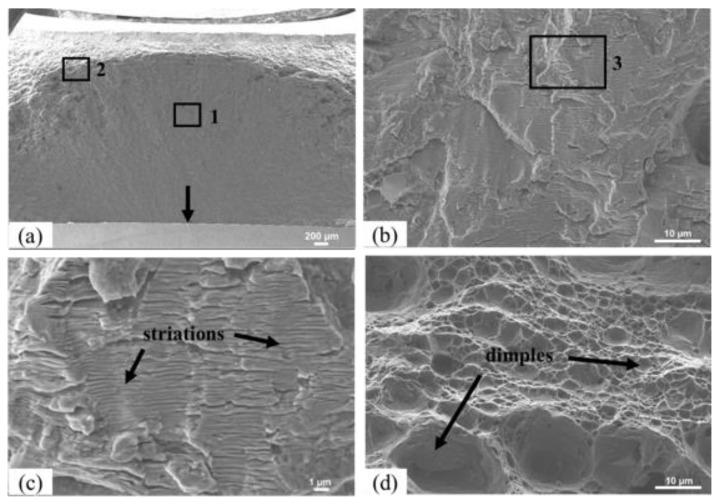
Fracture surfaces of a sample from the 562-30 series: (**a**) general fracture surface, (**b**) detail from location 1, (**c**) detail from location 3, and (**d**) detail from location 2.

The fracture surface of the fatigue specimens had identical morphology. Figure 16 illustrates the fracture surface of the specimen from the 562-30 series subjected to a stress range of 180 MPa that broke after 193,700 cycles. Figure 16a shows a general view of the fracture surface of the specimen, where the fracture zones analyzed in more detail are indicated with rectangles. The crack started on the welding surface marked with an arrow and was propagated by fatigue through the thickness of the skin. The orientation of the characteristic fatigue striations was perpendicular to the growth direction of the crack, as shown in Figure 16b,c in more detail. Figure 16c shows the detail marked with a rectangle in Figure 16b in greater magnification. In the final part of the crack propagation phase, a ductile fracture occurred, as evidenced by the presence of small dimples and some larger dimples in the transition zone, as seen in Figure 16d. The fracture of this test specimen occurred in the TMAZ on the advancing side of the AA5083 alloy.

## 4. Conclusions

The carried out research led to the following conclusions:

- It is feasible to achieve good quality FSWs with fillets between three dissimilar aluminum alloys.
- The nugget formation of FS welds between three dissimilar aluminum alloys is greatly influenced by the welding speed, mechanical properties, and location of the alloys, either on the advancing or retreating sides.
- A very low tool rotational to welding speed ratio (*w/v*) leads to the formation of welding defects.
- The weld nugget has a large dispersion of grain sizes, but the welding speed does not affect the grain size in the nugget.
- Increasing the welding speed increases the static and fatigue resistance of the welded joints.
- Placing the more resistant alloy (AA2017) on the advancing side rather than on the retreating side generates higher local weld temperature and provides stronger joints and with better fatigue behavior.

## Figures and Tables

**Table 1:** Chemical composition of the base materials (wt.%).

Alloy	Cu	Mg	Mn	Si	Cr	Others
AA2017-T4	4.5	0.8	0.7	0.8	≤0.1	Bal.
AA5083-H111	0.025	4.5	0.57	0.09	0.25	Bal.
AA6082-T6	0.09	0.7	1.0	0.53	<0.25	Bal.

**Table 2:** Mechanical properties of the tested aluminum alloys.

Properties	AA2017-T4	AA5083-H111	AA6082-T6
Ultimate Tensile Strength (MPa)	416.8 ± 3	317.5 ± 5.8	344.6 ± 5.7
Tensile Yield Strength (MPa)	293 ± 9.2	145 ± 4.1	286 ± 13.4
Elongation at Break (%)	18 ± 3.9	22.7 ± 1.4	18.4 ± 1.6
Vickers Hardness (HV_0.2_)	116.7 ± 4.2	82.3 ± 1.4	115 ± 0.8

**Table 3:** Welding parameters used to make the three dissimilar T-joints.

Material Position	Series	V (mm/min)	*w/v* (r/mm)
562	562-30	30	16.7
	562-120	120	4.2
	562-280	280	1.8
265	265-30	30	16.7
	265-120	120	4.2
	265-230	230	2.2

**Table 4:** Chemical composition in the various zones of both the 562-30 and 265-30 series (wt.%).

Welds Series	Zone	Mg	Si	Cu	Material Composition
562-30	Z1	0.66	0.45	1.75	2017
	Z2	0.81	0.79	0.14	2017/5083/6082
	Z3	2.3	0.57	0.4	2017/5083/6082
	Z4	3.32	0.32	0.37	5083/2017
	Z5	4.85	…	…	5083
	Z6	0.72	0.64	0.4	2017/6082
	Z7	1.29	0.51	0.6	2017/5083/6082
265-30	Z1	0.6	0.7	0	6082
	Z2	4.3	0.6	4.7	2017/5083/6082
	Z3	1.2	…	3	2017/5083
	Z4	0.6	0.8	1.6	2017/6082

**Table 5:** Average tensile results of defect-free welded specimens.

Weld Series		Ultimate Tensile Strength (MPa)	Efficiency (%)	Strain (%)	Fracture Zone
562	562-30	273.3 ± 0.5	86.0	10.6 ± 1.8	HAZ
	562-120	277.4 ± 0.4	87.4	8.3 ± 2.6	HAZ
265	265-30	271.7 ± 0.4	85.6	8.9 ± 0.4	HAZ
	265-120	296.2 ± 4.2	93.3	8.2 ± 2.5	HAZ
